# Intermediate service input distortions and total factor productivity: Evidence from China

**DOI:** 10.1371/journal.pone.0296429

**Published:** 2024-01-02

**Authors:** Meng Shen, Tian Liu

**Affiliations:** Economic Department, Capital University of Economics and Business, Beijing, China; Gabriele d’Annunzio University of Chieti and Pescara: Universita degli Studi Gabriele d’Annunzio Chieti Pescara, ITALY

## Abstract

China has the most minimal proportion of intermediate service inputs among the input-output datasets encompassing 43 countries and regions within the WIOD database. This study employs a non-parametric estimation methodology to compute the appropriate input levels for two distinct categories of intermediate goods. Furthermore, it evaluates the decline in total factor productivity resulting from distortions in intermediate input. The research findings are as follows: 1) China’s producer services exhibit an output elasticity approximately twice that of industrial intermediate goods. However, the input for producer services is only about half of that for the latter. This points to a notable deviation of China’s input for producer services from the optimal level. 2) Upon achieving an optimal level of input allocation for intermediates, the entire industry could experience an 11.48% boost in total factor productivity. In particular, the manufacturing sector could witness an impressive surge of 33.91%. 3) A positive correlation is discerned between intermediate input distortions and the import of intermediate products.

## Introduction

**Fig 1:**
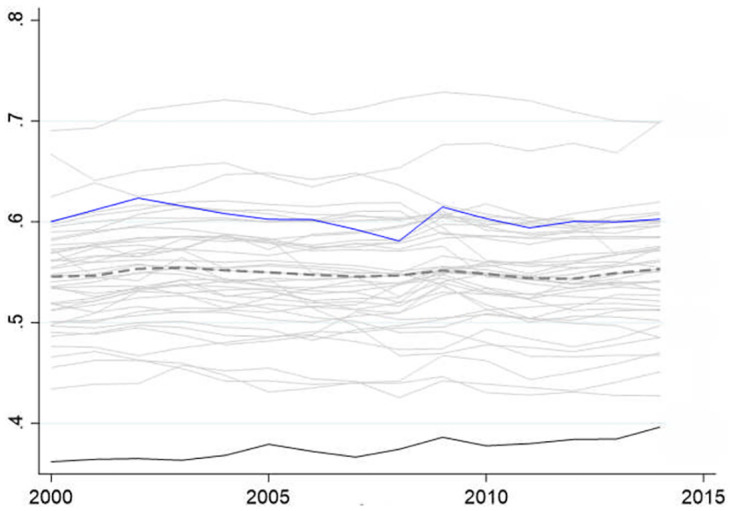
Intermediate service inputs as a share of total intermediate inputs. (A) Fig 1 shows the annual average of intermediate service inputs for the 43 countries in the WIOD database, China ranks lowest in 43 countries for intermediate service inputs. (B) The dotted line is the average share of world inputs in intermediate services, the blue line is the United States, the solid black line is China, and the gray line is for the other 41 countries.

Intermediate services, encompassing domains like design, research and development, finance, and logistics, play a pivotal role as intermediary inputs. These services predominantly find their place in the upstream sectors of industries, dictating the trajectory of China’s economic restructuring and the metamorphosis of its economic growth model. Based on data from the World Input-Output Database (WIOD), it’s evident that China’s average utilization of intermediate services as inputs across industries accounted for 37.48% during the period spanning from 2000 to 2014. This proportion not only falls below the 56.70% average observed in developed nations like those within the OECD but also notably deviates from the global average of 54.88% (Fig 1). Consequently, China stands at the bottom among the 43 countries and regions covered in the database.

The glaring disparity in the utilization of intermediate service inputs in China starkly contrasts with its remarkable economic progress, marking a significant deviation from global trends. This inconsistency underscores the urgent need for a more in-depth investigation into this matter. The term ’de-industrialization’ is intricately linked to this phenomenon, portraying a process where the relative importance of manufacturing declines while the share of services increases [1]. A substantial body of literature has sought to elucidate this phenomenon by focusing on the growing fragmentation of national manufacturing value chains and the gradual outsourcing of service activities from the manufacturing core [2–7]. This paper diverges from previous approaches that analyze restructuring and structural change within the manufacturing sector, as we provide insights into the relationship between intermediate inputs and total factor productivity.

Assessing whether the presence of intermediate service inputs leads to losses in total factor productivity confronts intricate challenges, primarily rooted in endogeneity concerns. According to conventional economic theory, in the absence of market barriers, the cost-return ratios of diverse input factors are expected to converge. When the marginal rate of return on intermediate service inputs exceeds that of other input factors, it becomes logical to escalate the usage of intermediate services to amplify the overall output. However, it’s crucial to note that conventional measures may introduce estimation biases [8]. For instance, augmenting industrial intermediate inputs might yield more pronounced benefits for technological advancement, potentially leading to an underestimation of the advantages conferred by industrial intermediate inputs, especially when total factor productivity isn’t directly observable.

Building upon insights from De Loecker and Warzynski [8], this paper commences by meticulously computing unbiased output elasticities for two distinct types of intermediate goods: industrial intermediates and intermediate services. Subsequently, employing the cost-benefit maximization principle, it calculates the ratio of output elasticities to expenditure shares for each type of intermediate, wherein the disjunction between the two ratios signifies the extent of distortion in intermediate service inputs. This culminates in the quantification of total factor productivity losses attributable to distortions in intermediate service inputs.

This paper introduces an innovative non-parametric estimation methodology situated within the framework of monopolistic competition. The aim is to estimate the marginal rate of return for factors while sidestepping potential endogeneity issues that could arise. This approach provides a more comprehensive and accurate assessment of the role played by intermediate inputs, addressing the intricacies introduced by factors such as technological progress. By employing this approach, the study enhances the credibility of its findings, providing valuable insights into the allocative efficiency of intermediate inputs and their impact on total factor productivity within China’s economic landscape.

The subsequent sections of this study are structured as follows: The literature review section reviews the literature related to this paper. The theoretical model section outlines a model designed for gauging the output elasticity of both industrial intermediate goods and intermediate services within the industry. The data section describes the data sources and processing. We then discussed the results of the empirical investigation. The section of futher discussion expands the analysis to consider the impact on total factor productivity within the external market context. The section of the conclusion encapsulates the key findings of the study. The final section discusses the limitations encountered in the study and suggests directions for future research.

## Literature review

The ascendancy of the division of labor in global value chains has prompted an increasing emphasis on research concerning intermediate inputs. Recent years have seen a notable focus on trade in intermediate goods, technological spillovers originating from these goods, and the diminishing distinctions between manufacturing and services [9–12]. A substantial portion of global trade is characterized by the exchange of intermediate goods, with developed countries often exporting specialized intermediate inputs to developing nations and importing final products [9]. Numerous studies confirm the role of productivity enhancement through the utilization of imported intermediate inputs [13–15].

In recent years, there has been a notable trend of outsourcing manufacturing activities to service providers, leading to a gradual blurring of the boundaries between manufacturing and services. The relocation of manufacturing to developing countries, particularly at the lower end of the value chain, has raised concerns among scholars about the "de-industrialization" of developed countries [2, 7]. However, in the case of China, the scenario is characterized by low inputs of productive services and high inputs of industrial intermediates.

Two primary factors contribute to the limited investment in intermediate services in China. Firstly, when viewed from an industrial chain perspective, highly developed industrialized nations have undergone significant structural shifts towards service-oriented societies, with the industrial sector gradually moving to emerging and developing nations [16]. China’s trajectory, driven by market-oriented reforms since the 1980s, has integrated it into the international division of labor system. However, China’s integration has mainly been positioned at a "low-end embedded" level [17]. Presently, China’s externally focused economic development is predominantly characterized by OEM production and processing trade, resulting in severed links between manufacturing and productive services [18]. Importantly, the latter lacks the necessary market support stemming from intermediate demand in the manufacturing sector. This deficiency in demand for intermediate services has consequently led to a scarcity of intermediate service inputs in China.

China is deeply integrated into the global value chain through its involvement in processing and manufacturing activities. However, the responsibilities associated with managing the upstream and downstream links related to intermediate services are predominantly shouldered by other countries. As a result, the demand for intermediate services within firm production in China remains limited. Neely highlights that China stands out in their sample, as it is a nation where manufacturing companies predominantly sell goods without engaging substantially in service provision [19]. The proportion of service firms in China relative to the total number of firms is a mere 0.97%, a stark contrast to the U.S. figure of 58.57%. Miroudot and Cadestin discern that around 50% of the value of China’s exported goods can be attributed to foreign service inputs, underscoring the significance of these inputs [20]. Koopman et al., investigating industries with substantial high-tech content such as electronic equipment, reveal that approximately 80% of the value of goods originates from foreign services [21].

In emerging markets, the service sector tends to lag behind its developed counterparts due to inherent inefficiencies. Consequently, firms in these markets prefer to engage with more efficient imported services, sidelining the less efficient domestic service sector [22]. In contrast, developed nations actively intervene and fortify their manufacturing sectors through processes like manufacturing servitization, which includes activities such as maintenance, research and development, and design [23].

Secondly, the limited input of intermediate services in China can be attributed to supply capacity constraints. When examined from a market perspective, the growth of the service sector faces hindrances due to excessive protectionism and a lack of competition, resulting in overall inefficiency. Unlike the markets for final goods, the intermediate goods market requires a more robust institutional framework. The existing suboptimal institutional and market environment in emerging markets, coupled with insufficient service standardization, incurs elevated operational costs. Additionally, domestic regulations and trade barriers further impede the externalization and market integration of intermediate service industries, ultimately resulting in meager inputs for the production process. Significantly, intermediate services are inherently knowledge-intensive [24], encompassing domains such as R&D services, software, information services, e-commerce, and network communications. These services rely on advanced information technology and high-end technology. However, China’s indigenous supply capacity remains insufficient, necessitating a dependence on imported intermediate services.

The advancement of intermediate services, crucial for promoting both production coherence and efficiency, stands as a pivotal component in enhancing the international competitiveness of the manufacturing sector. However, in China, the prevailing trend leans towards the utilization of industrial intermediate goods, with relatively minimal consumption of intermediate services—especially those embedded with advanced technology. Extensive research has emphasized that intermediate services can significantly contribute to enhancing the competitiveness of manufacturing industries [25, 26]. Unfortunately, the prevalence of low-value-added intermediate inputs fosters a weakened capacity for technological innovation within Chinese enterprises, thereby impeding the ascent of the country’s manufacturing sector in the global value chain. It is essential to recognize that intermediate goods constitute a form of capital and wield considerable influence, accounting for approximately half of the total output [27].

Some literature has focused on the fact that part of the production process in manufacturing is being provided by service firms, and that many high-value-added activities are being outsourced to the service sector, particularly business services from external suppliers, as well as the skilled services of technicians [1, 28, 29], there has been insufficient research on the relationship between industrial intermediates, intermediate services, and productivity. This paper closely aligns with the literature exploring the impact of factor mismatches on total factor productivity. While existing research primarily delves into the allocative efficiency of labor and capital markets [30–32], the dimension of allocative efficiency of intermediate inputs has received limited attention. Donovan uncovers evidence of the repercussions of low intermediate inputs on total factor productivity within the agricultural sector [33]. Calthrop et al. assess the cost-effectiveness of transport inputs using a general equilibrium model that takes transport infrastructure services as its entry point [34]. Some scholars have even quantified the influence of financial frictions on total factor productivity [35, 36].

In this paper, we argue that two factors limit the research on input distortions of intermediate services. First, research in factor mismatch has generally evolved from Hsieh and Klenow(HK model) with micro firms [37]. While micro data are certainly characterized by fidelity and large samples, micro firm datasets that provide multidimensional and comparable intermediate inputs are relatively rare, and those that provide intermediate services in particular are even more scarce. Second, the flexibility and customizability of intermediate services make it difficult to apply the HK model, which measures factor market distortions based on differences in the prices or returns of "homogeneous factors" of different market players [38]. Distortions in capital and labor markets can be attributed to market inconsistencies, such as the differential treatment of capital between state-owned and private capital [39], which are manifested in the differences in prices or returns of homogeneous factors used by different market players. However, the market of intermediate services is deeply segmented, and there is considerable heterogeneity among different market players, which makes it difficult to measure the benefits of "homogeneous services" of different players.

The underrepresentation of intermediate service inputs in China is a recognized phenomenon within the existing literature [17, 40]. However, minimal attention has been directed towards examining this issue from the vantage point of allocative efficiency concerning intermediate goods. It is worth noting that firms possess the flexibility to choose various combinations of industrial intermediates and intermediate services for their intermediate goods inputs. Low levels of intermediate service inputs do not necessarily translate into a decline in total factor productivity if such choices stem from market-driven freedom. Conversely, when these inputs are constrained, they indeed contribute to diminished total factor productivity. Therefore, it becomes imperative to address the fundamental question of whether the low inputs of intermediate services in China result in a detrimental impact on total factor productivity.

## Theoretical model

In this paper, a non-parametric estimation method is used to measure the optimal input share of intermediate services with total factor productivity loss. First, the cost-benefit of factor inputs is discussed by transcending the logarithmic production function. Second, in estimating total factor productivity, since technological progress is related to intermediate inputs, which leads to possible endogeneity problems in the model, non-parametric estimation is used to calculate the output elasticity of each intermediate input and measure the optimal intermediate input share accordingly. Finally, the difference between the optimal output and the actual output corresponding to the time when each intermediate input share is optimal is estimated to obtain the total factor productivity loss.

### Production function construction

In this paper, we categorize intermediate goods used in the production process into two primary classifications: intermediate services and industrial intermediate goods. Industrial intermediate goods encompass a range of items such as electricity, energy, steel, cement, and building materials. On the other hand, intermediate services encompass financial services, transportation, and information technology, among others. These categories represent the diverse elements involved in the production process. To capture these concepts more explicitly, we denote industrial intermediate goods as *ind*_*it*_, and intermediate services as *serv*_*it*_. All intermediary inputs are collectively represented as *M*_*it*_. Assuming that industry i follows a production function at time t as described by Eq (1):

$$Y_{it}=A_{it}F(L_{it},K_{it},ind_{it},serv_{it})$$ (1)

where *L*_*it*_ is labor, and *K*_*it*_ represents capital. The production function is characterized by the following assumptions. Firstly, costs are attributed to labor and capital adjustments in contrast to intermediate goods. Labor and capital are treated as fixed inputs, while intermediate inputs are considered variable inputs. Secondly, we assume Hicks neutrality in technology, denoted as *A*_*it*_. Lastly, the production function is assumed to exhibit constant returns to scale, yet it accommodates intricate substitution or complementarity relationships among input factors, which are assumed to balance at a value of one over a particular timeframe.

Considering cost-effectiveness, optimal input selection leads to a scenario where the marginal rate of technological substitution aligns with the price ratio. This equivalence is also expressed as "the output elasticity ratio equals the input share ratio." Letting $s_{it}^{m}=\frac{P_{it}^{m}m_{it}}{P_{it}Y_{it}}$ represent the proportion of total income allocated to a specific intermediate input (termed the input share), and $\xi_{it}^{m}$ represents the output elasticity of intermediate good m, the relationship can be formulated as follows:

$$\frac{s_{it}^{\text{ind}}}{s_{it}^{\text{serv}}}=\frac{\xi_{it}^{\text{ind}}}{\xi_{\text{it}}^{\text{serv}}}$$ (2)

In a situation of optimal input allocation, the "output elasticity to share ratio" remains consistent across all intermediate goods, and it’s set equal to the industry’s markup rate. This is also the basic formula for the DLW method (De Loecker and Warzynsiki) to measure the markup rate. Although the DLW method implicitly assumes that the "output elasticity to share ratio" of each variable is equal, it has not been tested empirically. Raval uses five sets of commonly used microdata to test this and finds that the markup rates calculated using labor and materials are not only unequal but also negatively correlated [41].

However, in cases where there are hidden market barriers, the optimal distribution might diverge from this equilibrium. For instance, despite the lower cost associated with intermediate services, the presence of elevated search costs could prompt firms to opt for an alternative strategy by "internalizing" these services and enlarging their in-house service divisions. Consequently, this choice would cause shifts in the output elasticity-to-share ratio for both categories of intermediate goods.

### Estimation of total factor productivity

To assess the output elasticity of intermediate inputs, the initial step involves estimating the production function. In this study, we employ the advanced logarithmic production function model to replicate the comprehensive production function. This is accomplished by introducing quadratic terms for the input factors along with interaction terms, as illustrated in Eq (3):

$$y_{it}=\gamma_{1}m_{it}+\gamma_{2}m_{it}^{2}+\gamma_{3}k_{it}+\gamma_{4}k_{it}^{2}+\gamma_{5}l_{it}+\gamma_{6}l_{it}^{2}+\gamma_{7}m_{it}k_{it}+\gamma_{8}m_{it}l_{it}+\gamma_{9}l_{it}k_{it}+\omega_{it}+\varepsilon_{it}$$ (3)

where *ω*_*it*_ is total factor productivity, *ε*_*it*_ is the random error term, and the parameter vector *γ* is the vector to be estimated. Total factor productivity might be intertwined with input factors, resulting in the well-recognized challenge of the simultaneity problem concerning input factors and total factor productivity [42]. Given that both categories of intermediate inputs have an impact on total factor productivity, this situation introduces bias into production functions derived from conventional estimation methods. To derive a reliable estimate of the production function, it becomes imperative to account for the latent productivity shock term.

Consequently, to achieve a coherent estimation of the production function, this study employs a non-parametric estimation methodology. This approach effectively manages the interference caused by unobservable total factor productivity disturbances and facilitates the generation of unbiased production function estimates. In theoretical terms, both industrial intermediate goods and intermediate service intermediate goods can be formulated as functions of additional factor inputs and the technological level.


$$m_{it}=h^{m}\left(L_{it},K_{it},\omega_{it}\right)$$ (4)

Proxy variables necessitate the existence of a one-to-one functional correlation with productivity to facilitate the derivation of the inverse function. In the context of this study, total factor productivity exhibits complexity, encompassing not only the overall technological level but also accounting for the detriment arising from deviations of intermediate inputs from cost minimization. Consequently, the functional linkage between intermediate goods and total factor productivity does not necessarily meet the requisite conditions. To mitigate the ensuing bias, this paper adopts two types of intermediate goods (namely, industrial intermediate goods and intermediate services) as proxies for total factor productivity in the initial step of the regression. This strategy serves to mitigate the endogeneity issue stemming from potential market distortions.

Given the stipulation that labor and capital are treated as fixed inputs within this study, the determination of total factor productivity involves using the two classifications of intermediate goods inputs as proxies. Consequently, the process enables the calculation of productivity through an inversion of the equation: *ω*_it_ = (*L*_it_, *K*_it_, *m*_it_). Total factor productivity is computed using the following procedure:

In the first step, an unbiased estimate ${\hat{y}}_{it}$ of the total output is estimated, as shown in Eq (5).


$${\hat{y}}_{it}=y_{it}-\varepsilon_{it}$$ (5)

Since the missing productivity leads to endogeneity problems in the model, the estimated parameters need to be corrected by a second step. The second step is to construct the General Method of moment (GMM). Let ***Z*** represent the set consisting of various types of inputs, at which point total factor productivity can be expressed as follows.


$$\omega_{it}={\hat{y}}_{it}-\gamma_{it}Z_{it}$$ (6)

Given a parameter vector *γ*, *ω*_*it*_ is the total factor productivity of each industry i at moment t given *γ*, i.e., the residual value after deducting the contribution of factors such as the number of factor inputs to economic growth. Drawing on the approach of Olley and Pakes (1996), total factor productivity *ω*_*it*_ is assumed to obey a Markov process, where total factor productivity in the current period is only affected by the previous period. Then we have

$$\omega_{it}=g\left(\omega_{it}|\omega_{it-1}\right)+\sigma_{it}$$ (7)

In Eq (7), g is represented by a third-order polynomial of *ω*_*it*-1_ as an explanatory variable of *ω*_*it*_, and *σ*_*it*_ is a random perturbation term. Thus, the vector composed of explanatory variables is $X_{it-1}=(1,\omega_{it-1},\omega_{it-1}^{2},\omega_{it-1}^{3})$. Let the number of samples be N and ***ω***_*it*−1_ represent the vector composed of the total factor productivity of all samples in the previous period with the number of elements N. Then $\omega_{it-1}^{2}$ and $\omega_{it-1}^{3}$ represent the column vectors composed of the squared and cubed terms of the total factor productivity of each industry i at moment t-1, respectively. Let the vector ***β*** = (*β*_0_, *β*_1_, *β*_2_, *β*_3_)′, then we have

$$\beta ={\left(X\prime_{it-1}X_{it-1}\right)}^{-1}X\prime_{it-1}\omega_{it}$$ (8)

Where *X*′ represents the matrix transpose, where the **β** solution process is similar to the parameter solution of multiple regression, and *σ*_*it*_(*γ*) is calculated as follows

$$\sigma_{it}(\gamma )=\omega_{it}-X_{it-1}\beta$$ (9)

Capital and labor are assumed here to be fixed inputs, with quantities determined in the previous period and unaffected by shocks. This is mainly because changes in capital and labor require adjustment costs and are more lagged compared to intermediate goods. Based on the nature of variable input intermediate goods m being independent of lagged 1-period productivity, the GMM conditions for the parameters can be obtained, whereby an unbiased production function can be obtained as follows.


$$E\left[\sigma_{it}(\gamma ){\left(m_{it-1},k_{it},m_{it-1}^{2},l_{it}^{2},k_{it}^{2},m_{it-1}l_{it},m_{it-1}k_{it},l_{it}k_{it}\right)}^{\prime }\right]=0$$ (10)

### Calculation of total factor productivity loss

According to Eq (3), the output elasticity of intermediate inputs can be calculated by first-order partial derivatives, as shown in Eq (11). Accordingly, $\xi_{it}^{m}$ is the output elasticity of input m.


$$\xi_{it}^{m}={\hat{\gamma }}_{1}+2{\hat{\gamma }}_{2}m_{it}+{\hat{\gamma }}_{7}k_{it}+{\hat{\gamma }}_{8}l_{it}$$ (11)

The optimal input share of intermediate services is calculated as follows:

$$\left(s_{it}^{serv^{*}}=\frac{\xi_{it}^{serv}}{\xi_{it}^{ind}+\xi_{it}^{serv}}\right)$$ (12)

The actual intermediate input share is calculated from $m_{it}/{\hat{y}}_{it}$, according to which the output elasticity and share ratio of the two types of intermediate goods can be calculated.

Total factor productivity is affected when the actual share of intermediate inputs differs from the optimal share. After the production function is obtained empirically, the optimal shares of different intermediate inputs can be theoretically measured by planning solutions, $s_{it}^{*}=\left(s_{it}^{ind*},s_{it}^{serv*}\right)$. The optimal output corresponding to the optimal share is denoted as ${\hat{y}}_{it}^{*}$

$${\hat{y}}_{it}^{*}=\gamma_{1}m_{it}^{*}+\gamma_{2}{\left(m_{it}^{*}\right)}^{2}+\gamma_{3}k_{it}+\gamma_{4}k_{it}^{2}+\gamma_{5}l_{it}+\gamma_{6}l_{it}^{2}+\gamma_{7}m_{it}^{*}k_{it}+\gamma_{8}m_{it}^{*}l_{it}+\gamma_{9}l_{it}k_{it}$$ (13)

Then the theoretical total factor productivity loss is the difference between optimal and actual output, as shown in Eq (14).


$$\Delta \omega_{it}={\hat{y}}_{it}^{*}-{\hat{y}}_{it}$$ (14)

If the share of a certain input is always lower than its proper share, there is an efficiency loss, i.e., the expected value of total factor productivity *E*_*i*∈*N*_[Δ*ω*_*it*_]. Due to the presence of distortions in intermediate inputs, there exists a theoretical potential to enhance output without augmenting costs. This can be achieved by reallocating industry expenditures to procure additional intermediate inputs that are currently priced below their optimal level. Consequently, this loss in output can be perceived as a reduction in total factor productivity. Naturally, the underlying source of these input distortions may be the concealed expenses triggered by market barriers. In the absence of rectifying these market impediments, industries encounter challenges in attaining optimality through internal adjustments.

## Data

The data presented in this paper have been acquired from the World Input-Output Database (WIOD) and the Socio-Economic Accounts (SEA) tables, both of which are provided by the World Input-Output Database. The most recent iteration of the WIOD table encompasses input-output information for 56 industries across 43 countries or regions, spanning from 2000 to 2014. Additionally, the Socio-Economic Accounts (SEA) table furnishes data about labor, capital, intermediate inputs, value added, and overall output for each industry within each country. These datasets facilitate the assessment of intermediate goods’ utilization within the production process, as well as the analysis of value added and total output for each industry.

Following the guidelines of the United Nations’ International Standard Industrial Classification (ISIC), Fourth Edition, the WIOD table comprises 3 agricultural, 24 manufacturing, and 29 service industries. After eliminating 9 industries that displayed significant deficiencies, the resultant sample comprises 3 agricultural industries, 23 manufacturing industries, and 21 service industries. In the process of calculating intermediate goods, considering that the agricultural industry includes agricultural services, agricultural intermediate goods are counted as intermediate services to ensure the integrity of intermediate inputs. To ensure robustness, we excluded agricultural intermediate goods and did a test on the later results, which did not change significantly.

Due to constraints in sample availability, this study opts to utilize input-output data from 47 industries in China spanning the period from 2000 to 2014, culminating in a total of 705 samples. To commence the analysis, the paper allocates the intermediate goods inputs for each industry, as dictated by the WIOD table. This allocation encompasses manufacturing-originating intermediate goods as industrial intermediate inputs and service-originating intermediate goods as intermediate services. Furthermore, imported intermediate goods are also factored into the analysis.

During the calculation of intermediate goods, it is noted that the agricultural industry incorporates agricultural services. To ensure the completeness of intermediate inputs, agricultural intermediate goods are categorized as intermediate services. To validate the robustness of the analysis, agricultural intermediate goods are excluded, yielding consistent results. In a subsequent step, all data are converted into U.S. dollars based on the market exchange rate for the respective year. Finally, nominal indicators related to intermediate inputs and total output are adjusted using the price index of intermediate goods and the price index of final goods. This adjustment standardizes the indicators to constant 2000 prices.

In terms of industry scale, the manufacturing sector significantly surpasses the services sector in terms of total output (*Y*), labor (*L*), and capital (*K*). As illustrated in Table 1, the cumulative inputs for industrial intermediate goods (*Ind*) at the industry level exceed those for intermediate services (*Serv*). It becomes evident that within individual industries, the input of industrial intermediate goods in the manufacturing sector far outweighs that of intermediate services. In contrast, the service industry showcases a nearly equal magnitude of industrial intermediate input and intermediate services input.

**Table 1:** Descriptive statistical analysis.

Variables	Whole Industry	Manufacture	Service
Y	11.9198 (1.2522)	12.2680 (1.2294)	11.5862 (1.1826)
L	8.6436 (1.5280)	8.8270 (1.2950)	8.4523 (1.7036)
K	11.4191 (1.5150)	11.7051 (1.1199)	11.1449 (1.7733)
Ind	10.8521 (1.5014)	11.7352 (1.2657)	10.0058 (1.1906)
Serv	10.2552 (1.2078)	10.3897 (1.2028)	10.1263 (1.2001)
*Import*_*int*_	0.0773 (0.0419)	0.0872 (0.0473)	0.0333 (0.0679)
*Import*_*serv*_	0.0452 (0.0274)	0.0494 (0.0300)	0.0411 (0.0239)
*Import*_*ind*_	0.0957 (0.0529)	0.9537 (0.0530)	0.0960 (0.0529)
samples	705	345	360

Table 1 reports the mean and standard deviation of labour, capital, industrial intermediate goods and intermediate services for China’s manufacturing and service sectors, as well as for all industries.

^a^ The standard deviation is in parentheses in the table, and the mean of each variable is above the parentheses.

In addition, from the perspective of imports, intermediate goods account for 7.73% of the total intermediate input in the entire industry(*Import*_*int*_). Among them, the proportion of intermediate goods imports in the manufacturing sector is higher, at around 8.72%. The share of imported production services in its total input (*Import*_*serv*_) is not high, at around 4%. On the other hand, the proportion of industrial intermediate goods imports in its total input (*Import*_*ind*_) is approximately 9%, indirectly indicating that importing production services in the industry is relatively challenging. Descriptive statistics of the variables are shown in Table 1.

## Results and discussion

### Baseline regression results

As a baseline regression, the fixed effects method is initially employed to estimate the production function and the output elasticities of the two categories of intermediate inputs(For brevity, fixed effects regression results are not presented in the main text and are available from the S1 Table). This is done to examine potential distortions in intermediate input utilization within China. The estimation results of the output elasticities for the two types of intermediate goods are presented in Table 2 using the fixed-effects method. In this study, it is assumed that intermediate inputs are linked to technological advancement. To preserve the integrity of the technological progress proxy function in subsequent analyses, we refrain from cross-multiplying the two types of intermediate goods in this section.

**Table 2:** Fixed effects result in output elasticities and optimal inputs for intermediate goods.

year	*∂Y*/*∂ind*	*∂Y*/*∂ser*	The share of optimal intermediate services s*	Actual share of intermediate services	Difference
2001	0.2982	0.2448	0.451	0.3644	0.0866***
2002	0.3064	0.2526	0.452	0.3650	0.0870**
2003	0.3069	0.2664	0.465	0.3635	0.1015***
2004	0.3105	0.2794	0.474	0.3682	0.1058***
2005	0.3215	0.3004	0.483	0.3793	0.1037***
2006	0.3311	0.309	0.483	0.3722	0.1108***
2007	0.3439	0.3206	0.482	0.3666	0.1154***
2008	0.3444	0.3337	0.492	0.3783	0.1137***
2009	0.3505	0.3493	0.499	0.3903	0.1087***
2010	0.3566	0.3535	0.498	0.3823	0.1157***
2011	0.3573	0.3652	0.505	0.3785	0.1265***
2012	0.3608	0.3781	0.512	0.3892	0.1228***
2013	0.3689	0.3879	0.513	0.3897	0.1233***
2014	0.3730	0.3984	0.516	0.4010	0.1150***

The results reported in Table 1 are all means.

^a^ The first column of Table 2 reports the industrial intermediate output elasticities for 2001–2014, the second column shows the intermediate services output elasticities, the third column represents the optimal intermediate service input share calculated based on Eq (12), and the fifth column shows the difference between the actual intermediate services input share and the optimal productive input share.

^b^ The significance level of the difference is the result obtained from Bootstrap, we randomly sampled 2,000 times to obtain 2,000 optimal intermediate service inputs, and the significance level was calculated by observing how often the actual intermediate service inputs were less than the optimal intermediate service inputs.

*** p<0.01,

** p<0.05,

* p<0.1.

The analysis reveals a gradual increase in the output elasticity of the industry-wide average utilization of industrial intermediate goods, ascending from 0.30 in 2001 to 0.37 in 2014. Similarly, the output elasticity of intermediate service inputs shows an upward trend, ranging from 0.24 to 0.40. The concurrent rise in the output elasticities of both intermediate goods categories suggests that technological progress indeed contributes to heightened intermediate goods utilization. This phenomenon may also indicate the presence of endogeneity in the fixed effects model.

Calculations based on optimization indicate an ideal proportion of around 50% for intermediate service inputs. However, the actual input share in China falls below 40%, signifying a deviation of approximately 10% from the optimal allocation. Furthermore, a statistically significant difference is identified between the actual and optimal input shares. To determine whether this discrepancy signifies distortion, a bootstrap self-sampling method is employed. The analysis assesses the probability that the optimal input share of intermediate services is lower than the actual input share. The results demonstrate statistical significance at the 5% level, confirming the existence of intermediate input distortion within China. It is worth noting that the results could still be biased due to the oversight of technological progress. As such, further utilization of non-parametric estimation is required to address the endogeneity issue stemming from technological progress.

### Endogenous problems

The outcomes of the fixed effects regression indicate a notable discrepancy between China’s proportion of intermediate services and the optimal share for such services. However, the explanatory variables no longer meet the assumption of strict exogeneity due to the unobservable nature of total factor productivity. To address this endogeneity concern, Table 3 presents the results after mitigating the issue through the application of the nonparametric estimation method.

**Table 3:** Output elasticities and optimal inputs for intermediate goods by a non-parametric method.

year	*∂Y*/*∂ind*	*∂Y*/*∂ser*	The share of optimal intermediate services s*	Actual share of intermediate services	Difference
2001	0.2776	0.6242	0.6922	0.3644	0.3278*
2002	0.2799	0.6267	0.6913	0.3650	0.3262*
2003	0.284	0.6354	0.6911	0.3635	0.3277*
2004	0.2873	0.6421	0.6909	0.3682	0.3226*
2005	0.2921	0.6501	0.6900	0.3793	0.3107*
2006	0.2957	0.6543	0.6888	0.3722	0.3165*
2007	0.2999	0.6590	0.6873	0.3666	0.3207*
2008	0.3022	0.6656	0.6877	0.3783	0.3094*
2009	0.3048	0.6708	0.6875	0.3903	0.2973*
2010	0.3072	0.6736	0.6868	0.3823	0.3045*
2011	0.3108	0.6814	0.6867	0.3785	0.3082*
2012	0.3128	0.6861	0.6869	0.3892	0.2977*
2013	0.3156	0.6898	0.6861	0.3897	0.2964*
2014	0.317	0.6929	0.6861	0.4010	0.2851*

The results reported in Table 1 are all means.

^a^ The first column of Table 2 reports the industrial intermediate output elasticities for 2001–2014, the second column shows the intermediate services output elasticities, the third column represents the optimal intermediate service input share calculated based on Eq (12), and the fifth column shows the difference between the actual intermediate services input share and the optimal productive input share.

^b^ The significance level of the difference is the result obtained from Bootstrap, we randomly sampled 1,000 times to obtain 1,000 optimal intermediate service inputs, and the significance level was calculated by observing how often the actual intermediate service inputs were less than the optimal intermediate service inputs. See S1 Fig for regression parameters.

*** p<0.01,

** p<0.05,

* p<0.1.

In comparison to the fixed effects model, the nonparametric estimation approach calculates lower output elasticities for industrial intermediate goods and relatively higher ones for intermediate services. This implies that technological progress exerts a more substantial influence on industrial intermediate goods, while intermediate services like finance and logistics remain more stable.

The pronounced shortfall in intermediate service inputs becomes even more apparent when the impact of technological progress is excluded. Specifically, the optimal share of intermediate service inputs in the total pool of intermediate goods should be around two-thirds. However, in reality, the share of intermediate goods input remains below 40%.

Confidence intervals to gauge the distortion in intermediate inputs are also computed through the Bootstrap self-sampling technique. In comparison to the fixed effects model, the confidence intervals derived from the nonparametric estimates are wider. Moreover, the original hypothesis of no difference is rejected at a significance level of 10%. Empirical findings from both the fixed effects and nonparametric estimation models consistently underscore the presence of distortions in Chinese intermediate inputs, reinforcing the robustness of this conclusion. It should be noted that the optimal share here is the local optimal solution. The author further relies on the planning solution calculation of the production function to obtain the global optimal solution, but the difference between the local optimal solution and the global optimal solution is small. Only when the production function is a constant elasticity of substitution (CES) production function, the two are exactly equal. This is due to the fact that the output elasticity changes again after a change in the share of intermediate inputs.

### Placebo test

In this paper, the non-parametric estimation method is formulated based on the approach developed by De Loecker and Warzynski [8]. However, this approach has faced criticism recently, particularly concerning the "output elasticity share ratio" when employing different inputs. Given these concerns, it becomes imperative to assess the robustness of this method.

As a general belief holds that the U.S. market is less distorted, characterized by relatively free choice among market players, and the allocation of the two types of intermediate inputs in industries is more reasonable, the U.S. data is utilized to scrutinize whether the conclusions drawn from this method align with reality. To ensure the solidity of the findings from this paper, a placebo test using U.S. data is conducted.

The non-parametric estimation technique is employed to ascertain the existence of distortions in intermediate inputs within the U.S. The outcomes are detailed in Table 4. Let $\eta_{it}^{\text{ser}}$ and $\eta_{it}^{\text{ind}}$ denote the ratios of output elasticity shares for intermediate services and industrial intermediate goods, respectively, while "*difference*" signifies the disparity between these ratios. The results reveal that the output elasticity share ratios for the two types of intermediate goods in the U.S., as measured by the nonparametric estimation method, are remarkably similar, both remaining relatively stable at approximately 1.5. Over time, there is a slight incremental trend. When these two ratios are equal, the output elasticity share ratio corresponds to the industry’s price markup level. An approximate markup level of 1.5 is well within a reasonable range, aligning with the core assessment of De Loecker and Eeckhout on markup rates in the U.S. [43].

**Table 4:** The ratio of output elasticities to shares for two intermediate goods in the United States.

year	$\eta_{it}^{ind}$	$\eta_{it}^{\text{serv}}$	Difference	year	$\eta_{it}^{ind}$	$\eta_{it}^{\text{serv}}$	Difference
2001	1.4943 (1.6159)	1.3379 (0.5406)	0.1564 (0.6472)	2008	1.5035 (1.7162)	1.5046 (0.7422)	-0.0011 (-0.0039)
2002	1.5955 (1.7053)	1.3031 (0.4656)	0.2923 (1.1763)	2009	1.8111 (2.3396)	1.4337 (0.5857)	0.3774 (1.1035)
2003	1.5856 (1.7195)	1.3409 (0.5582)	0.2447 (0.9463)	2010	1.6891 (2.1736)	1.4516 (0.5993)	0.2376 (0.7316)
2004	1.5879 (1.7565)	1.3892 (0.6130)	0.1987 (0.7345)	2011	1.6681 (2.1700)	1.4711 (0.5637)	0.1970 (0.6102)
2005	1.5823 (1.7546)	1.3911 (0.6130)	0.1913 (0.7056)	2012	1.8183 (2.5543)	1.4599 (0.5423)	0.3583 (0.9657)
2006	1.6658 (2.0113)	1.4161 (0.5968)	0.2497 (0.8197)	2013	1.7227 (2.2369)	1.4406 (0.5197)	0.2821 (0.8612)
2007	1.5851 (1.9868)	1.4373 (0.6190)	0.1477 (0.4867)	2014	1.7554 (2.4164)	1.4098 (0.4920)	0.3457 (0.9870)

^a^ The first column shows the ratio of output elasticity to share of industrial intermediate goods, the second column shows intermediate services, and the difference is the absolute value of the difference between the output elasticity share ratios of the two types of intermediate goods, with t-values in parentheses.

^b^ $\eta_{it}^{ind}$ and $\eta_{it}^{\text{serv}}$ are the output elasticity share ratios of industrial intermediate goods and intermediate services, respectively, with standard deviation in parentheses;

Moreover, a t-test is executed to evaluate the equality of means between the two ratios. The initial hypothesis, suggesting that intermediate service inputs are reasonable, remains unchallenged at the 10% significance level across all years. To augment the credibility of this outcome, a Bootstrap method is employed, self-sampling 1000 times. The results underscore that none of the years attains significance levels beyond 10%, reinforcing the notion that U.S. intermediate inputs are indeed reasonable.

The analysis of U.S. data indicates that the nonparametric estimation method utilized in this study provides unbiased estimates of U.S. intermediate inputs, suggesting that the computational bias associated with this approach is manageable. As a result, the robustness of the nonparametric estimation method employed to assess intermediate input distortions is affirmed, thereby enhancing the credibility of the findings in this study. Furthermore, this paper extends its measurement to other countries, uncovering that intermediate input distortions are generally smaller in OECD countries, whereas non-OECD countries demonstrate higher levels of distortion.

### Bootstrap results

**Fig 2:**
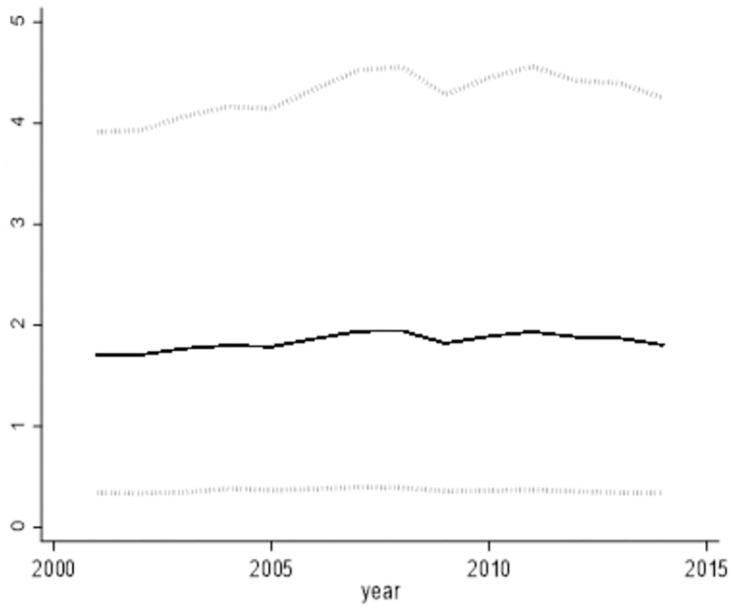
The Ratio of output elasticity of intermediate services to share of expenditure.

**Fig 3:**
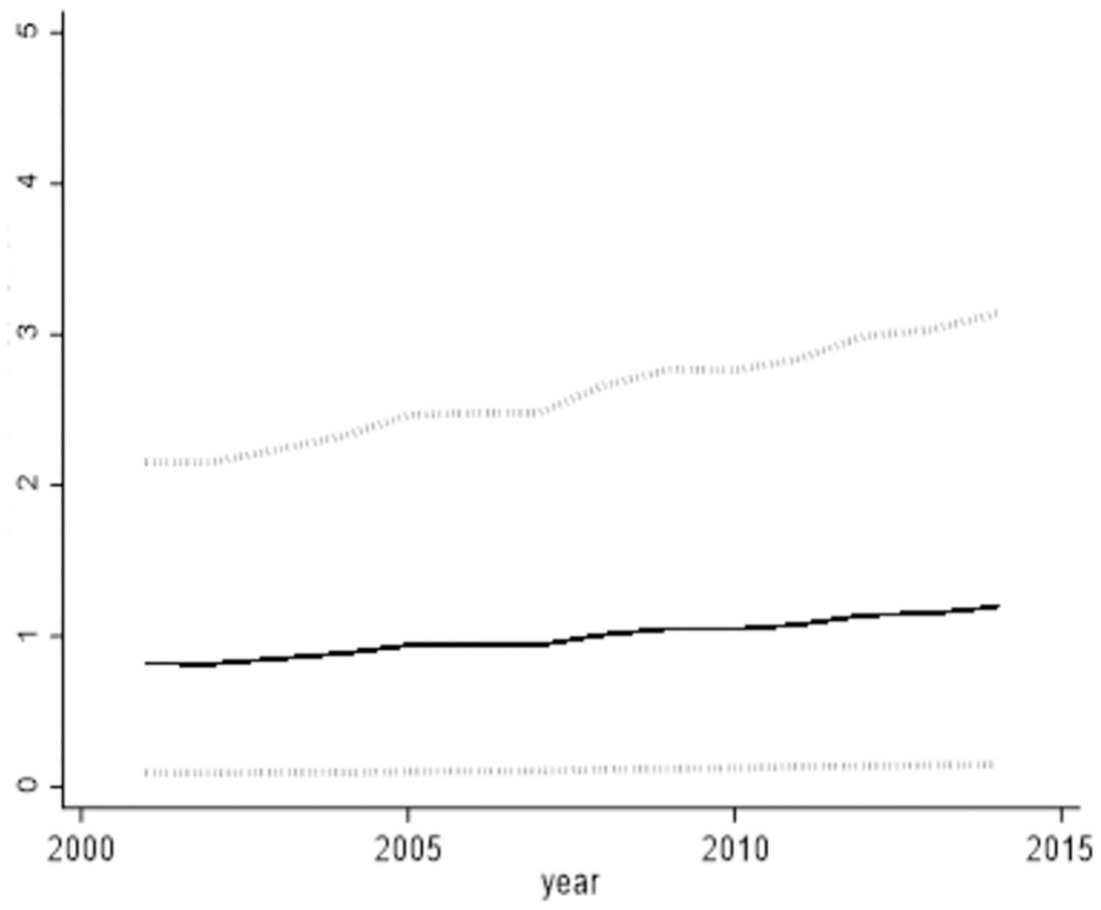
The ratio of output elasticity of industrial intermediate goods to expenditure share. (A) We conducted 1000 random samples from the dataset, resulting in 1000 outcomes. (B)The solid line in Figs 2 and 3 represents the 50th percentile of these 1000 outcomes, while the dashed lines correspond to the 5th and 95th percentile.

Through the effective resolution of the endogeneity challenge within the model, the findings of this paper are verified as robust. Figs 2 and 3 depicts the "output elasticity to share ratio" for the two categories of intermediate goods, as computed by the non-parametric estimation method. In theory, the "output elasticity to share ratio" corresponds to the "marginal revenue price ratio." Essentially, this ratio offers a cost-benefit evaluation of intermediate goods quantified in monetary terms. Inspecting the situation of industrial intermediate goods, the marginal output returns remained below 1 before 2007. This signifies that the value of the product obtained by investing 1 dollar in industrial intermediate goods is less than 1 dollar, This observation points to an excessive level of input for industrial intermediate goods.

Conversely, an additional input of 1 unit in intermediate service inputs yields a product value close to 2 units. This implies that the excessive marginal revenue price ratio for intermediate services signifies an inadequacy in intermediate service inputs. By the year 2014, across the nation’s 47 industries, an incremental unit of cost was invested in industrial intermediate goods inputs, resulting in a marginal return of approximately $1.02. In contrast, the marginal return on intermediate service inputs amounted to around $1.9. This suggests that the marginal return price ratio for industrial intermediate goods was about 50% of that observed for intermediate services.

### Sectoral heterogeneity

**Fig 4:**
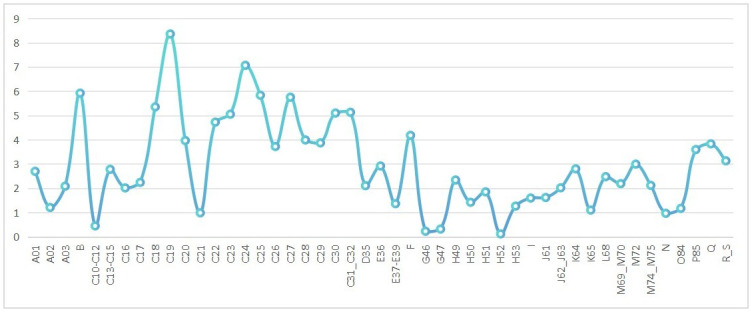
The difference in the ratio of output elasticity to share for two types of intermediate goods. (A)Values in the Fig 4 are industry averages for 2001–2014. (B)The specific industry information can be found in the S2 Table.

Fig 4 illustrates the differences in output elasticity and share for two types of intermediate goods across different industries. It can be observed that there are sectoral variations in the input distortion for the two types of intermediate goods. The top five sectors with the highest distortion levels are: Manufacture of coke and refined petroleum products, Manufacture of basic metals, Mining and quarrying, Manufacture of fabricated metal products, Manufacture of electrical equipment, and Manufacture of basic metals. The three sectors with the lowest distortion levels are: Warehousing and support activities for transportation, Wholesale trade, and Retail trade. Overall, the distortion level is higher in the manufacturing industry compared to the service industry.

**Fig 5:**
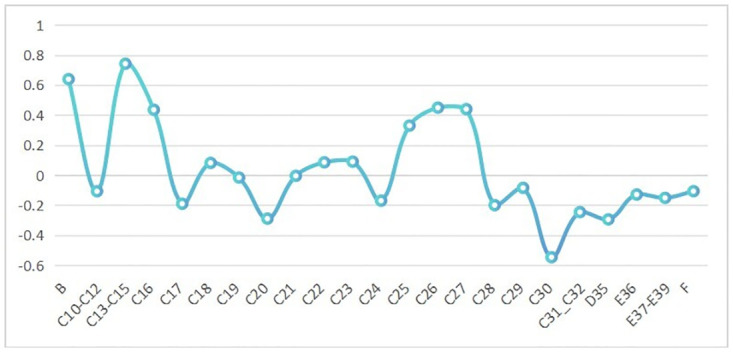
Correlation coefficient between KIBS input and manufacturing industry total factor productivity. (A) The specific industry information can be found in the S2 Table.

It is important to note that the service sector is broad, and the contributions to productivity vary across different service subsectors. Particularly, inputs from knowledge-intensive services play a crucial role in a high degree of innovation. Here, we calculate the relationship between KIBS (Knowledge-Intensive Business Services) inputs and industry total factor productivity for each sector, as shown in Fig 5. The input for KIBS (Knowledge-Intensive Business Services) originates from ISIC 64, 65 to 67, 71 to 74). KIBS inputs significantly enhance the productivity of manufacturing industries such as the manufacture of electrical equipment, manufacture of computer, electronic and optical products, mining and quarrying, and manufacture of textiles, wearing apparel, and leather products. In contrast, some sectors exhibit the opposite trend, such as Water collection, treatment, and supply sector, and electricity, gas, steam, and air conditioning supply.

### Total factor productivity loss

Based on the aforementioned calculations, it becomes evident that when the industry invests $1 in industrial intermediate goods, it garners a marginal return of less than $1. Conversely, investing $1 in intermediate services yields a marginal return of $2. This divergence in marginal revenue price ratios indicates an overinvestment in industrial intermediate goods. If a reallocation were to occur, diverting a portion of the expenditure from industrial intermediate goods to intermediate services, the industry’s overall output would increase. When both types of intermediate goods are distributed based on optimal inputs, the potential industry output can be established. The discrepancy between this potential output and the actual output represents the loss in total factor productivity.

**Fig 6:**
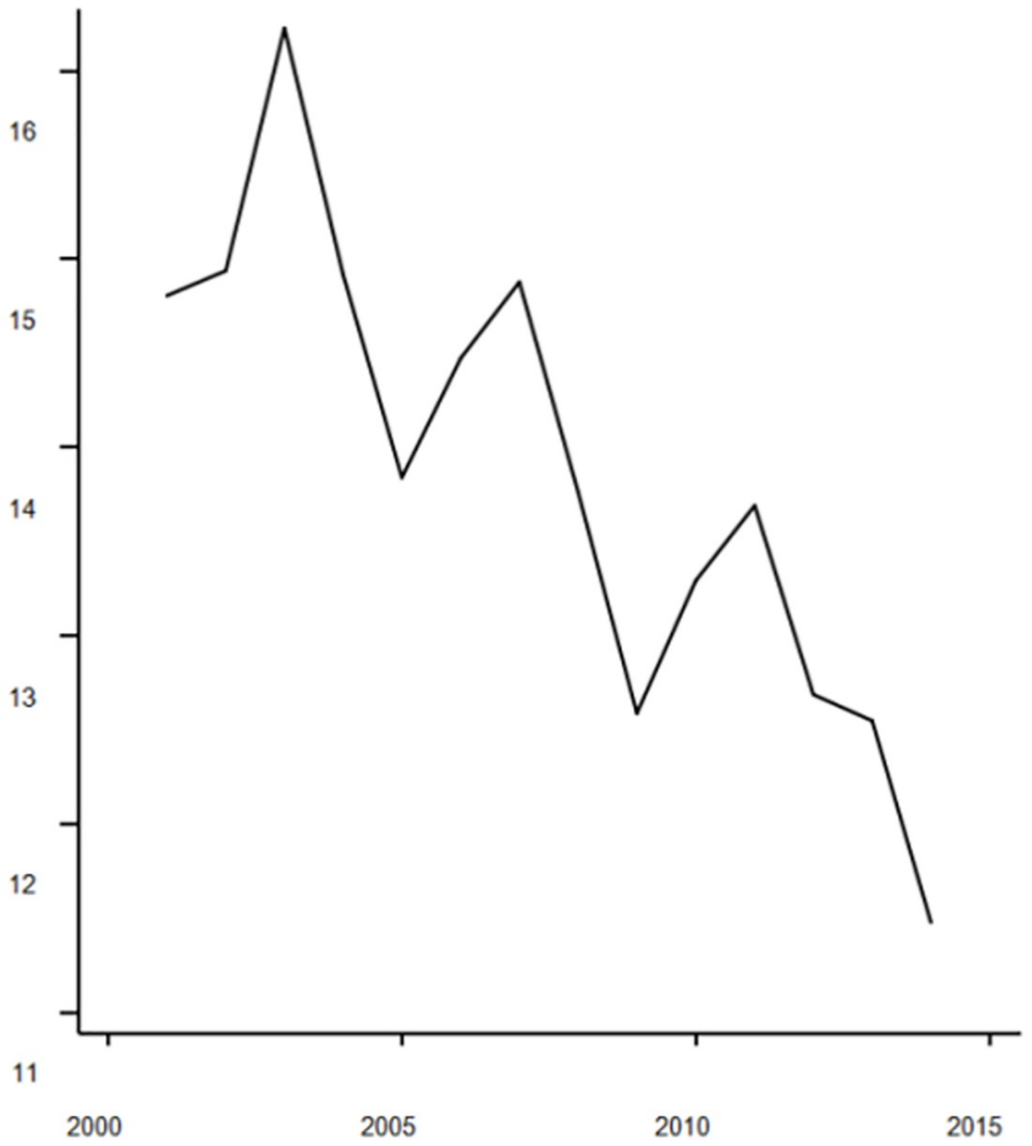
All industries’ total factor productivity loss.

**Fig 7:**
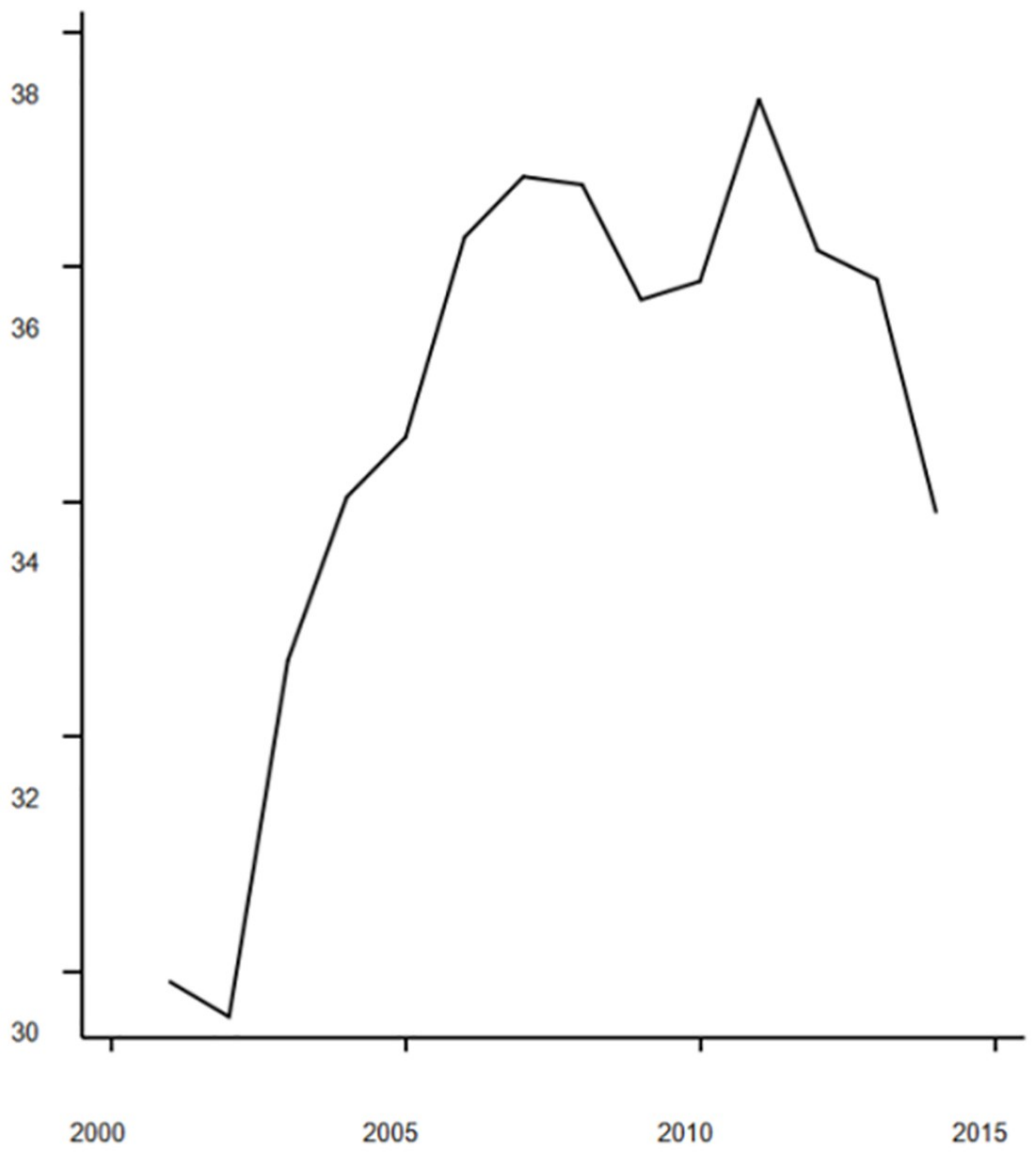
Total factor productivity loss in manufacturing. (A) Fig 6 shows the industry-wide average of total factor productivity losses from 2001–2014 and Fig 7 shows the average of total factor productivity losses in manufacturing from 2001–2014.

Fig 6 illustrates the industry-wide trajectory of productivity loss, showing a fluctuating downward trend. The highest loss was recorded at 16.22% in 2003, gradually declining to 11.48% by 2014. Analyzing from a sectoral standpoint, the Fig 7 presents total factor productivity losses within the manufacturing sector. This graph exhibits an initial increase followed by a subsequent decrease. Notably, heterogeneity exists in total factor productivity losses, with the manufacturing sector displaying greater losses compared to the overall sector. Optimizing the input share of intermediate services in manufacturing could result in a 33.91% productivity increase by 2014. On the other hand, the input share of the two intermediate goods types in the service sector approximates optimal levels by 2014.

## Further discussion

### Intermediate input distortion and intermediate goods import

The correlation between output elasticity and input share for both categories of intermediate goods highlights distortions in intermediate inputs within China, particularly with intermediate service inputs being significantly underestimated. In situations where market barriers hinder the utilization of intermediate services, companies might resort to substituting other intermediate goods, resulting in deviations from optimal input proportions. It’s important to note that, in cases of domestic market barriers for intermediate services, firms can also consider import substitution. Research has indicated that nations with limited domestic service development can mitigate barriers by importing intermediate goods from more developed countries [22].

Theoretically, intermediate input distortions should exhibit a positive relationship with the proportion of imported intermediate goods. Table 5 delves into the connection between intermediate input distortions and the import share of intermediate goods. The outcomes reveal a notable and significant positive correlation between the two factors. Upon comparing columns (2) and (3), it becomes apparent that the impact of intermediate input distortions on the import share of industrial intermediate goods lacks significance. Conversely, the import share of intermediate services demonstrates a substantial positive correlation, implying that the influence of intermediate input distortions on intermediate goods imports primarily originates from intermediate services. Column (4) proceeds to validate the robustness of these findings through an analysis involving imports of intermediate goods, and consistent results are obtained.

**Table 5:** Intermediate input distortions and imports of intermediate goods.

variables	(1) *Import*_*int*_	(2) *Import*_*ind*_	(3) *Import*_*serv*_	(4) *Import*
*dev*	0.0037*** (3.2352)	0.0019 (1.2245)	0.0025** (2.5812)	0.0325** (2.4244)
Control variables	Yes	Yes	Yes	Yes
Industry fixed effects	Yes	Yes	Yes	Yes
Sample size	658	658	658	658
*R* ^*2*^	0.3969	0.4214	0.2319	0.9008

^a^ "*dev*" represents the deviation in the elasticity of output between two types of intermediate goods and the ratio of their shares.

^b^ Control variables include labour, capital, industrial intermediate goods, intermediate services, the ratio of output elasticity of industrial intermediate goods, and intermediate services to share of expenditure.

This pattern elucidates that when distortions in intermediate inputs exist, industries tend to explore alternative factors of production for substitution. Simultaneously, they enhance imports of intermediate goods to acquire high-quality intermediates from the global market.

### Input distortions, imports and total factor productivity

Table 5 underscores a clear positive relationship between intermediate input distortions and imports of intermediate services. This raises the question: can imports of intermediate goods contribute to enhanced productivity? The findings are detailed in Table 6, showcasing the regression results of total factor productivity against imported intermediate goods. The calculation of total factor productivity employs the non-parametric estimation method discussed earlier.

**Table 6:** Intermediate input distortions, imports of intermediate goods and total factor productivity.

variables	(1) *ω*_*it*_	(2) *ω*_*it*_	(3) *ω*_*it*_	(4) *ω*_*it*_	(5) *ω*_*it*_	(6) *ω*_*it*_	(7) *ω*_*it*_
*Import*	1.0401*** (3.1341)				1.3514*** (4.0074)		
*Import*_*ind*_		0.1306 (0.4900)				0.2708 (1.0099)	
*Import*_*serv*_			1.8250*** (4.8885)				2.0566*** (5.5075)
*dev*				-0.0266*** (-3.0548)	-0.0349*** (-3.9452)	-0.0281*** (-3.1793)	-0.0341*** (-3.9545)
control variables	Yes	Yes	Yes	Yes	Yes	Yes	Yes
Industry fixed effects	Yes	Yes	Yes	Yes	Yes	Yes	Yes
Year fixed effects	Yes	Yes	Yes	Yes	Yes	Yes	Yes
Sample size	658	658	658	658	658	658	658
*R* ^*2*^	0.4879	0.4931	0.4858	0.5339	0.5399	0.5358	0.5376

^a^ Control variables include labour, capital, industrial intermediate goods, intermediate services, Ratio of output elasticity of industrial intermediate goods, and intermediate services to share of expenditure.

In Table 6, column (1) illustrates the regression of total factor productivity on imports of intermediate goods, revealing a positive relationship between such imports and total factor productivity. By comparing the outcomes for imports of industrial intermediate goods in column (2) and imports of intermediate services in column (3), it becomes evident that imports of intermediate services exert a more substantial impact on total factor productivity. However, the influence of imports of industrial intermediate goods is relatively limited. This disparity can be attributed to the maturity of the domestic market for industrial intermediate goods, where minimal distinction exists between the output elasticity of imported and local products. Conversely, the output elasticity of imported intermediate services surpasses that of local products, signifying that a higher import share is advantageous for bolstering total factor productivity.

Columns (4) to (7) introduce the intermediate input distortion variable. A comparison of coefficients before ’dev’ in the intermediate input distortion variable demonstrates that the impact of intermediate input distortion on total factor productivity is the weakest in column (4) when imports are not considered. Importantly, the negative impact of distortion on total factor productivity escalates after accounting for imports, implying that imports can alleviate intermediate input distortion to a certain extent.

The positive impact of imported intermediate goods on total factor productivity is also magnified when comparing results without accounting for distortions. For instance, the coefficient representing the import share of all intermediate goods on total factor productivity rises from 1.04 to 1.35. Importantly, even after controlling for ’*dev*’, the influence of the import share on total factor productivity continues to rise. This underscores that imports of intermediate goods can effectively optimize the allocation of intermediate inputs, ultimately enhancing total factor productivity in the context of China.

## Conclusion

This study employs global input-output data to compute the optimal distribution of input shares for two distinct types of intermediate goods, with a focus on the relative contribution of each factor input to an industry’s cost structure. Additionally, it estimates the resultant loss in total factor productivity arising from the distortion of intermediate inputs. The ensuing conclusions are as follows: Firstly, the output elasticity of intermediate services surpasses that of industrial intermediate goods twofold. Ideally, the input share for intermediate services should be twice that of industrial intermediate goods. However, in actuality, the input for intermediate services is only half that of industrial intermediate goods. This substantial discrepancy from the optimal allocation highlights a significant divergence in input distribution. This conclusion remains robust even after accounting for sample selection and endogeneity concerns, underscoring the presence of intermediate input distortions within the Chinese context. Secondly, the distortion of intermediate inputs leads to an industry-wide total factor productivity loss of 16.22%. By the year 2014, the manufacturing sector’s total factor productivity could be amplified by 33.91% if the input share of intermediate services is elevated to its optimal level. Thirdly, there exists a positive correlation between intermediate input distortions and imports of intermediate goods. Notably, the influence of intermediate input distortions on imports primarily stems from the domain of intermediate services. Lastly, industries grappling with more pronounced intermediate input distortions tend to adopt a strategy of importing intermediate goods. This proactive import behaviour effectively mitigates the extent of intermediate input distortions, subsequently enhancing overall total factor productivity.

In essence, this paper unveils the dissonance between ideal and actual input distributions for intermediate goods, shedding light on their consequential impact on total factor productivity. The findings highlight the role that imports play in ameliorating distortions and elevating productivity levels within industries. The recommended strategies in this paper are as follows:

Firstly, eliminate institutional barriers hindering the circulation of services. The inherent flexibility and customization within the productive service industry, coupled with the intricate nature of market access and qualification processes, have resulted in a notable deficiency in effective domestic supply. To counter this, a prudent regulatory approach is recommended. The government should consider reasonably relaxing market access, fostering diverse participation in service supply, intensifying competition in the intermediate goods market, and invigorating overall market dynamics. Enhancing the market environment for productive services is crucial, encouraging the unimpeded flow of production factors and commodity services across industries and regions. This entails optimizing production factor allocation, concentrating on the effective linkage of the industrial chain’s upstream, midstream, and downstream, and facilitating the openness of production, distribution, circulation, and consumption links.

Secondly, the paper advocates for the profound integration of the service industry with the manufacturing sector. Presently, China’s manufacturing and service industries confront issues of imbalanced development and weak collaboration, primarily evident in the manufacturing sector’s suboptimal selection of intermediate inputs, resulting in a staggering loss of total factor productivity (33.91%). This impedes the manufacturing industry’s utilization of productive services. Hence, concerted efforts should be directed at enhancing coordination between these sectors, and establishing a new system characterized by high quality, efficiency, and competitiveness within the service industry. The goal is to establish a dynamic equilibrium where manufacturing industry demands drive effective productive service industry supply, and the innovation within productive services elevates the quality of manufacturing industry development.

Lastly, the paper emphasizes the imperative to elevate the level of trade liberalization in intermediate goods. Importing intermediate goods offers a substantial remedy to the distortions in productive services. Active maintenance and enhancement of the multilateral trading system are vital, fostering a higher degree of openness. Encouraging international cooperation in productive services, active participation in global competition, and incentivizing enterprises to import productive services to create high-quality intermediate inputs are pivotal. This approach aims to steer the industrial division of labor towards the peaks of the "smile curve."

## Limitations and future research

It is essential to acknowledge the limitations of this study. Constrained by available data, our research covers the period from 2000 to 2014. Unfortunately, we were unable to explore the recent performance of intermediate inputs and total factor productivity, especially in the context of the challenges posed by the COVID-19 pandemic in China. Furthermore, this dissertation exclusively focuses on the correlation between intermediate inputs and total factor productivity within the Chinese context, lacking international comparative analyses—a crucial aspect, particularly in the context of production fragmentation.

Future research efforts should seek to validate the relationship between intermediate inputs and total factor productivity in China by incorporating more recent data. This should be accompanied by comparative analyses involving multiple countries, including but not limited to India, Korea, Japan, and the United States, along with OECD nations. Additionally, investigating the impact of external factors such as import and export trade on intermediate inputs and total factor productivity would present a valuable avenue for future research.

## Supporting information

S1 Table Fixed effects regression results. (PDF)

S2 Table Industry description and code. (PDF)

S1 Fig Kernel density plots of coefficients under the non-parametric estimation method. (PDF)
