# Supplementary material for: Intermediate service input distortions and total factor productivity: Evidence from China
Source: PLoS One. 2024 Jan 2;19(1):e0296429. doi: 10.1371/journal.pone.0296429 (PMC10760905; doi:10.1371/journal.pone.0296429)
Supplement: S1 Table — (PDF) [file pone.0296429.s001.pdf]

S1 Table provides the production functions for China and the United States calculated by the fixed-effects approach, and the output elasticities for the two types of intermediate goods are obtained from the derivation.

S1 Table. Fixed effects regression results

	(1)	(2)
VARIABLES	China	America
Ind	0.1585**	0.0000
	(2.1222)	-1.33
Ser	-0.7485***	0.6513
	(-6.8341)	5.58*
L	0.7695***	0.5091
	(7.8361)	5.18*
K	0.8219***	0.0000
	(11.3216)	-2.62*
ind*l	0.0665***	-0.1292
	(10.0844)	-22.9*
ind*k	0.0493***	-0.0083
	(5.1819)	-0.98
ser*l	0.0012	0.0985
	(0.1750)	5.48*
ser*k	-0.0025	-0.1288
	(-0.4940)	-8.79*
ind*ind	-0.0931***	0.0582
	(-9.7349)	13.65*
ser*ser	-0.0405***	0.0315
	(-4.1062)	2.7*
l*l	0.0305**	0.0057
	(2.1910)	0.66
k*k	-0.0182	0.0683
	(-1.5079)	10.84*
l*k	-0.0008	-0.0084
	(-0.1132)	-0.92
Constant	2.1357***	1.7409
	(7.3042)	3.92*
R^2	0.9854	0.9904
Year fixed effect	Yes	Yes
Industry fixed effect	Yes	Yes
Observation	658	658

Note: * * *, * * and * indicate the significance levels of 1%, 5% and 10%, respectively
