# Supplementary material for: Intermediate service input distortions and total factor productivity: Evidence from China
Source: PLoS One. 2024 Jan 2;19(1):e0296429. doi: 10.1371/journal.pone.0296429 (PMC10760905; doi:10.1371/journal.pone.0296429)
Supplement: S2 Table — (PDF) [file pone.0296429.s002.pdf]

Supporting information

S2 Table. Industry description and code

Industry	Code
Crop and animal production, hunting and related service activities	A01
Forestry and logging	A02
Fishing and aquaculture	A03
Mining and quarrying	B
Manufacture of food products, beverages and tobacco products	C10-C12
Manufacture of textiles, wearing apparel and leather products	C13-C15
Manufacture of wood and of products of wood and cork, except furniture; manufacture of articles of straw and plaiting materials	C16
Manufacture of paper and paper products	C17
Printing and reproduction of recorded media	C18
Manufacture of coke and refined petroleum products	C19
Manufacture of chemicals and chemical products	C20
Manufacture of basic pharmaceutical products and pharmaceutical preparations	C21
Manufacture of rubber and plastic products	C22
Manufacture of other non-metallic mineral products	C23
Manufacture of basic metals	C24
Manufacture of fabricated metal products, except machinery and equipment	C25
Manufacture of computer, electronic and optical products	C26
Manufacture of electrical equipment	C27
Manufacture of machinery and equipment n.e.c.	C28
Manufacture of motor vehicles, trailers and semi-trailers	C29
Manufacture of other transport equipment	C30
Manufacture of furniture; other manufacturing	C31-C32
Electricity, gas, steam and air conditioning supply	D35
Water collection, treatment and supply	E36
Sewerage; waste collection, treatment and disposal activities; materials recovery; remediation activities and other waste management services	E37-E39
Construction	F
Wholesale trade, except of motor vehicles and motorcycles	G46
Retail trade, except of motor vehicles and motorcycles	G47
Land transport and transport via pipelines	H49
Water transport	H50
Air transport	H51
Warehousing and support activities for transportation	H52
Postal and courier activities	H53
Accommodation and food service activities	I
Telecommunications	J61

Computer programming, consultancy and related activities; information service activities	J62-J63
Financial service activities, except insurance and pension funding	K64
Insurance, reinsurance and pension funding, except compulsory social security	K65
Real estate activities	L68
Legal and accounting activities; activities of head offices; management consultancy activities	M69_M70
Scientific research and development	M72
Other professional, scientific and technical activities; veterinary activities	M74_M75
Administrative and support service activities	N
Public administration and defence; compulsory social security	O84
Education	P85
Human health and social work activities	Q
Other service activities	R_S
