# Supplementary material for: Intermediate service input distortions and total factor productivity: Evidence from China
Source: PLoS One. 2024 Jan 2;19(1):e0296429. doi: 10.1371/journal.pone.0296429 (PMC10760905; doi:10.1371/journal.pone.0296429)
Supplement: S1 Fig — (PDF) [file pone.0296429.s003.pdf]

Since the use of non-parametric methods is only able to calculate the elasticity values for each type of input, and the parameter t-value is not available, we obtained 2000 results by sampling the sample 2000 times through the BOOTSTRAP method, and displayed the kernel density plots for each parameter in Figure A.2

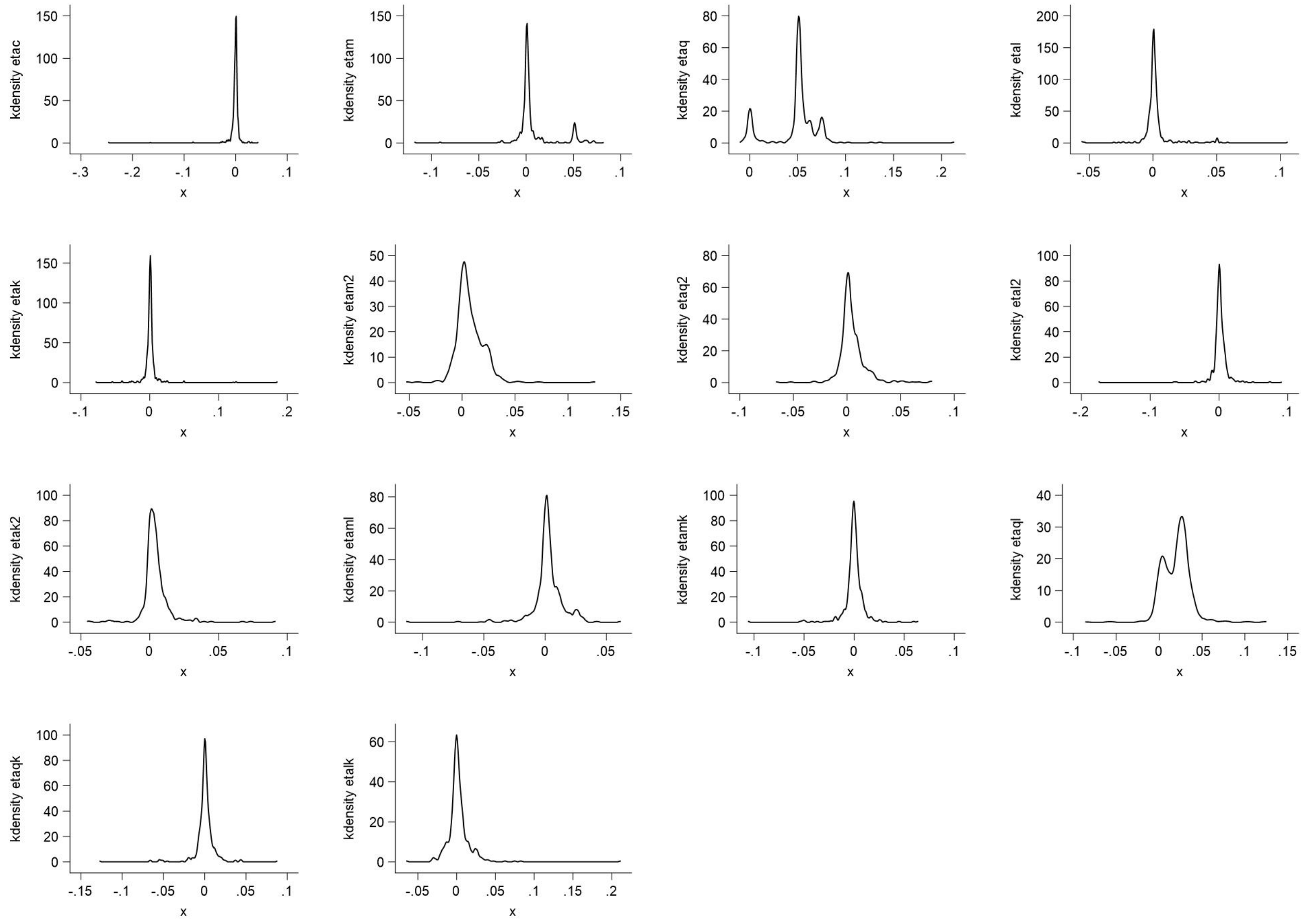

S1 Fig. Kernel density plots of coefficients under the non-parametric estimation method

Note: The parameters in the figure are in order from left to right and from top to bottom: c, ser, ind, l, k, ser*ser, ind*ind, l*l, k*k, ser*l, ser*k, ind*l, ind*k, l*k.
